# Dr. S. Jean Stevenson Monroe

**Published:** 1913-06

**Authors:** 


Dr. S. Jean Stevenson Monroe, Women's Medical College of Pennsylvania, 1870 (1885 according to State Directory), died at Bemus Point, N. Y., January 4, aged 71, of rectal cancer.

